# Interactions of Human Autoantibodies with Hippocampal GABAergic Synaptic Transmission – Analyzing Antibody-Induced Effects *ex vivo*

**DOI:** 10.3389/fneur.2015.00136

**Published:** 2015-06-11

**Authors:** Holger Haselmann, Luise Röpke, Christian Werner, Albrecht Kunze, Christian Geis

**Affiliations:** ^1^Hans Berger Department of Neurology, Jena University Hospital, Jena, Germany; ^2^The Integrated Research and Treatment Center for Sepsis Control and Care (CSCC), Jena University Hospital, Jena, Germany

**Keywords:** autoantibody, stereotactic injection, hippocampus, patch-clamp recording, limbic encephalitis, GAD65, GABAergic inhibition

## Abstract

Autoantibodies (aAB) to the presynaptic located enzyme glutamate decarboxylase 65 (GAD65) are a characteristic attribute for a variety of autoimmune diseases of the central nervous system including subtypes of limbic encephalitis, stiff person-syndrome, cerebellar ataxia, and Batten’s disease. Clinical signs of hyperexcitability and improvement of disease symptoms upon immunotherapy in some of these disorders suggest a possible pathogenic role of associated aAB. Recent experimental studies report inconsistent results regarding a direct pathogenic influence of anti-GAD65 aAB affecting inhibitory synaptic transmission in central GABAergic pathways. We here provide a method for direct evaluation of aAB-induced pathomechanisms in the intact hippocampal network. Purified patient IgG fractions containing aAB to GAD65 together with fixable lipophilic styryl dyes (FMdyes) are stereotactically injected into the hilus and the dentate gyrus in anesthetized mice. Twenty-four hours after intrahippocampal injection, acute hippocampal slices are prepared and transferred to a patch-clamp recording setup equipped with a fluorescence light source. Intraneural incorporated FMdyes show correct injection site for patch-clamp recording. Whole-cell patch-clamp recordings are performed from granule cells in the dentate gyrus and extracellular stimulation is applied in the border area of the dentate gyrus-hilus region to stimulate GABAergic afferents arising from parvalbumin positive basket cells. GABA-A receptor mediated inhibitory postsynaptic currents (IPSC) and miniature IPSC are recorded after blocking glutamatergic transmission. This approach allows investigation of potential aAB-induced effects on GABA-A receptor signaling *ex vivo* in an intact neuronal network. This offers several advantages compared to experimental procedures used in previous studies by *in vitro* AB preincubation of primary neurons or slice preparations. Furthermore, this method requires only small amounts of patient material that are often limited in rare diseases.

## Introduction

IgG autoantibodies (aAB) to the glutamate decarboxylase 65 (GAD65) are increasingly recognized in neurological diseases of the central nervous system, e.g., in stiff person-syndrome (SPS) (1, 2), limbic encephalitis (3), Batten’s disease (4), or cerebellar ataxia (5). GAD65 is the rate limiting enzyme for GABA synthesis in presynaptic nerve endings of GABAergic interneurons (6). Signs of hyperexcitability or reduced central inhibition in these disorders point toward a potential pathogenic role of the associated aAB to GAD65 (7, 8). However, there are several concerns arguing against direct pathogenic mechanisms induced by the patient’s aAB to GAD65. First, clinical syndromes are diverse and different regions and networks in the CNS seem to be afflicted, e.g., the hippocampus and amygdala in the subgroup of anti-GAD65 aAB positive limbic encephalitis, the brainstem and spinal cord in SPS, or the cerebellum in cerebellar ataxia (5). Second, GAD65 is an intracellular located enzyme that is less likely accessible to aAB compared to antigens on the neuronal surface. Third, in many of these syndromes exists a concurrent immune response to a variety of neuronal antigens in addition to GAD65. In SPS, patients with aAB to GAD65 develop often additional AB to the GABA-A receptor associated protein (GABARAP) (9). In Batten’s disease, aAB to several antigens are described, e.g., to alpha-fetoprotein and further, still unknown neuronal antigens (10). So, if there are specific pathogenic mechanisms induced by aAB, it is uncertain which of the disease associated aAB causes disease symptoms. There are several experimental studies investigating patient derived IgG targeting GAD65 in neurons in different settings (8, 11, 12). However, results obtained from these studies are heterogeneous and sometimes contrasting. *In vitro* studies using dissociated neuronal cell cultures reported reduced GABAergic inhibitory synaptic transmission upon preincubation with patient IgG containing anti-GAD65 aAB (13). In a recent study, we could provide evidence patient IgG derived from patients with SPS induced disturbance of GABAergic transmission. However, this was not caused by associated aAB to GAD65 but by IgG to another, yet unknown presumably presynaptic antigen that is included in the IgG fraction of these patients (14). In animal studies, patient IgG with high titers of aAB to GAD65 was reported to induce disturbed GABAergic inhibition in the spinal cord, cerebellum, or cortical areas (8, 15, 16). However, the target-specificity of IgG-induced pathophysiology to GAD65 remains to be shown. This can be achieved by use of recombinant, specific IgG derived from isolated human plasma cells in animal passive-transfer models as shown before for aAB to aquaporin 4 in mouse models for neuromyelitis optica (17, 18). Another approach is the use of affinity-purified IgG aAB extracted of the polyclonal patient IgG fraction (19–21).

However, patient-derived material is often limited because many of these syndromes are rare diseases with only few affected patients. Invasive interventions required obtaining patient material, e.g., lumbar punction for cerebrospinal fluid (CSF) cannot be performed repetitively in those patients for ethical reasons.

Here, we propose a method to investigate direct aAB induced effects *ex vivo* after stereotactic application of patient IgG preparations into the hippocampal compartment using only very limited amounts of patient material. We describe the possibility of histological and electrophysiological analyses of GABAergic pathways in this passive-transfer mouse model.

## Methods

### Purification of antibody-containing IgG fractions

IgG of an example patient with SPS and high titers of anti-GAD65 antibodies in serum and CSF as well as control IgG fractions without specific antineuronal reactivity were purified from therapeutic plasma exchange material by separation on exchange chromatography. Clinical details of the patients have been reported previously (16). The IgG fraction was concentrated by passage of the eluate through a Dialflo ultrafilter membrane (YM 100; Amicon, Witten, Germany) under nitrogen pressure to a volume of 50 ml. After dialysis against 10 l of water, the IgG sample was freeze-dried and stored at −20°C. Before use, lyophilized IgG was dissolved in 0.9M saline to a concentration of 5 mg/ml (16, 20).

### Stereotactic injections of patient IgG fractions into the hippocampus of mice

**Figure 1:**
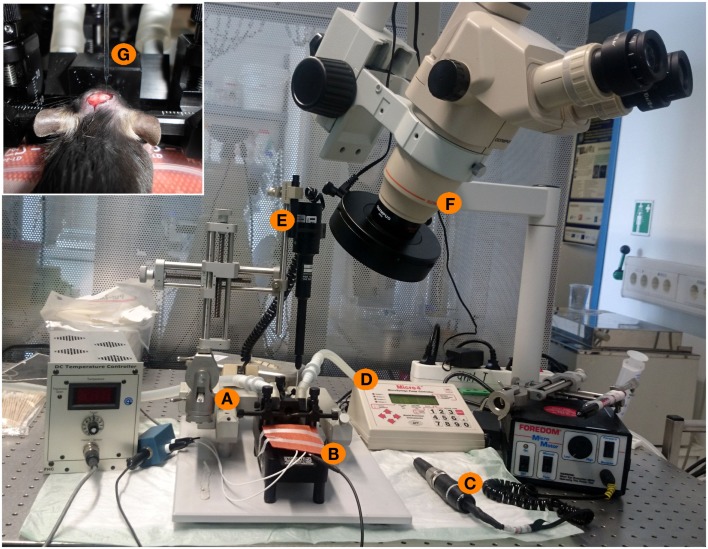
**Experimental setup for stereotactical injections of patient IgG fractions into the mouse hippocampus**. **(A)** Stereotactic frame with two arms (one for fixation of the injection pipette, one for fixation of a marker). **(B)** Heating pad with a feedback mechanism and a rectal probe to maintain the physiological temperature of 36°C of the mouse during surgery. **(C)** Dentist driller with a drill head of 0.47 mm diameter. **(D,E)** Micro4-controller for injection of nanoliter volumes with the injection pipette. **(F)** Binocular for optical control of all experimental steps. **(G)** Inset showing the mouse skull during surgery. Intracranial holes are already drilled. The upper part shows the glass injection pipette with the fine taper and filled with oil and IgG solution during injection into one of two holes of the mouse skull.

All animal experiments have been approved by the Thuringian state authorities (authorization # 02-059/13). Before surgery, the injection glass pipette (Glass Capillaries for Nanoliter 2000; Order# 4878; WPI, Sarasota, FL, USA) has been pulled with a long taper using a micropipette puller (Micropipette Puller P-1000; Sutter, Novato, CA, USA). The tip was then cut to a diameter of about 10 μm with a fine scissor. The injection pipette was attached to the head of a microprocessor-controlled nanoliter injector (Nanoliter 2000+ SYS-Micro4 Controller; WPI, Sarasota, FL, USA) and slightly filled with immersion oil to assure consistent pressure release onto the fluid by the injector. Thereafter, the pipette was completely filled with purified patient IgG fractions containing aAB against GAD65 (protein concentrations of 5 mg/ml in 0.9M saline). Together with the IgG fractions, 1 mM of FM1-43FX-dye (life technologies, Darmstadt, Germany) was added for fluorescent labeling of the area that has been targeted by the stereotactic intrahippocampal injection. A 6- (electrophysiological experiments) or 8-week-old (other experiments) wild type C57BL/6 mice were anesthetized with 1.5–2.0% isoflurane/oxygen and their head was fixed to a stereotactic apparatus (Figure 1; Lab StandardTM; Stoelting, Wood Dale, IL, USA). The temperature of the animals was monitored rectally and adjusted to 37°C by a heating pad with feedback control (FHC, Bowdoin, Canada). Eye ointment was used to prevent corneal drying during the surgery and the head was shaved with a razorblade. The skull was exposed by a small midline skin incision and the stereotactic injection sites were marked according to the respective coordinates (Table 1) determined according to the Paxinos mouse atlas (22). Holes were carefully drilled into the skull with a dentist driller (Foredom, Bethel, CT, USA) using drill heads of 0.47 mm diameter (Harvard Apparatus, Cambridge, UK) under visual control with an Olympus stereomicroscope (Olympus SZ 60, Tokyo, Japan). Care was taken not to penetrate the dura to avoid injury of the first cortical layers. For visual control of injection volumes, the initial maximum fluid level in the pipette was marked before each injection. The injection pipette was carefully moved to the respective injection point according to the coordinates (Table 1; Figure 1). After waiting 1 min, 1 μl of purified patient IgG solution together with fixable lipophilic styryl dyes (FMdyes) was injected into each hole with an injection speed of 4 nl/s. If the injection of solutions failed because the pipette was clogged by brain tissue, the injection pipette was removed and the injection was repeated with a new injection pipette. In some cases, minimal dorsal-ventral movement of the injection pipette was sufficient to perform proper injection without changing the pipette. After injection, the pipette was left in place for additional 5–10 min, before slowly being withdrawn. Thereafter, the skin was sutured with surgical suture (Covidien Sofilk 3-0, Dublin, Ireland) and the animals were monitored on a heating pad until complete recovery from anesthesia. All animals were sacrificed 1–4 days after surgery for immunohistological or electrophysiological experiments.

**Table 1:** **Coordinates for injections of IgG preparations in different parts of the hippocampal formation in mice (from bregma in mm)**.

Target	Anterior-posterior	Medial-lateral	Distal-ventral
Middle dentate gyrus (front end)	2.2	1.2	2.0
Middle dentate gyrus (tale end)	2.2	2.0	2.0
End dentate gyrus (front end)	2.5	1.5	2.0
End dentate gyrus (tale end)	2.5	2.5	2.5
Middle CA1 region	2.2	2.0	1.5
End CA1 region	2.5	2.3	2.0

**Figure 2:**
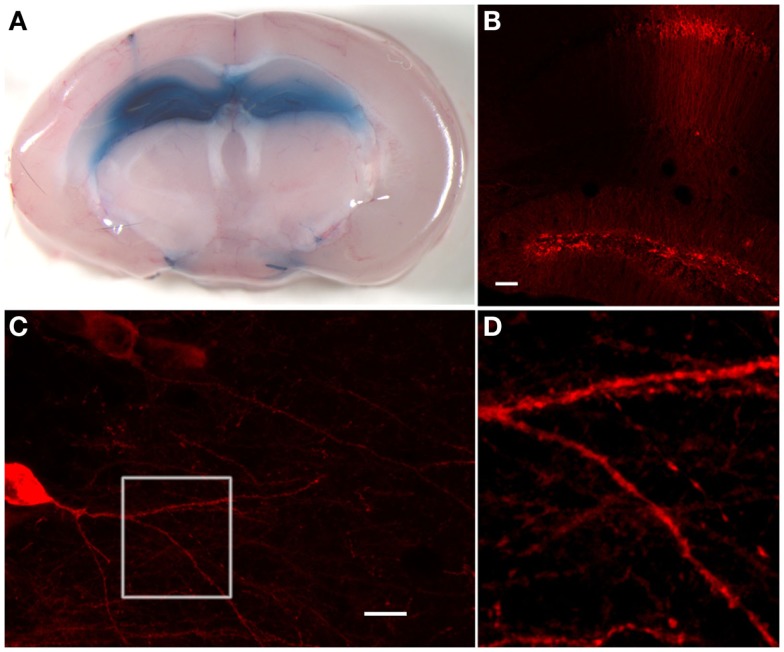
**Hippocampal stainings after stereotactic injections of trypan blue or anti-GAD65 aAB containing IgG preparations**. **(A)** Injection of 1 μl trypan blue in the dentate gyrus of each hemisphere shows uniform distribution exclusively in the hippocampal compartment. On the left side, the injection channel can be identified. **(B)** Immunohistological staining of injected anti-GAD65 aAB containing IgG preparation shows specific staining of cells in the dentate gyrus and around the injection channel (scale bar: 100 μm). **(C)** Dendrites of a CA1 pyramidal cell can be analyzed after injection of anti-GAD65 aAB containing IgG preparations. The magnification of dendrites at the branching region of a CA1 neuron [white box shown in **(D)**] shows staining of human IgG deposits with distinct spots distributed along the dendrite (scale bar: 20 μm).

In a subset of animals, we injected 1 μl of a 0.4% trypan blue solution (Sigma-Aldrich, St. Louis, MO, USA) in the same way as described above to verify correct injection sites of stereotactic injection procedure (Figure 2). Here, 2–4 h after injection, the animals were sacrificed and the brain was cut in 1 mm slices with a tissue chopper (McIlwain Tissue Chopper, Mickle Laboratory Engineering Co Ltd., Guildfort, UK) and the *ex vivo* slices were directly viewed under a stereomicroscope (Zeiss Stemi SV6, Zeiss, Jena, Germany).

### Immunohistology of IgG-injected brain slices

At 3–4 days post-injection, mice were deeply anesthetized, and intracardiac perfusion was performed. A catheter was placed into the left cardiac ventricle, and mice were perfused with 10 ml of phosphate buffered saline (PBS) followed by 4% paraformaldehyde (PFA) in phosphate buffer. Brains were extracted, afterfixated for 24 h in 4% PFA, dehydrated for 24 h in 10% sucrose, then further 24 h in 30% sucrose. Thereafter, 40 μm serial sections were prepared on a freezing sliding microtome (HM 450 Sliding Microtome; Thermo Scientific, Waltham, MA, USA). Slices were stored in anti-freeze solution (30% ethylene glycol, 700 mM glucose, 3 mM sodium azide in phosphate buffer, pH 7.4) at −20°C until the day of staining. Slices were then defrosted at room temperature and washed six times for 15 min in tris-buffered saline (TBS). Unspecific binding sites were blocked by 3% normal goat serum and 2% milk powder in TBS containing 0.1% of Triton-X100 for permeabilization of the membrane for 30 min. Slices were incubated with Cy3 goat anti-human antibodies (Dianova, Hamburg, Germany) as secondary antibodies to detect binding of the injected human aAB in 3% normal goat serum, 2% milk powder, and 0.1% TritonX-100 overnight at 4°C. After additional washing, steps in TBS for 10 min slices were placed on object slides, dried on air, stained for 5 min in DAPI solution (Sigma-Aldrich, St. Louis, MO, USA), washed three times in PBS for 5 min, and mounted with Fluoromount (Southern Biotech, Birmingham, AL, USA).

### Confocal microscopy

Cells were viewed on a confocal microscope (Zeiss LSM 710, Jena, Germany) using a 10× objective or a 63× oil objective, keeping laser power and photomultiplier tube voltage constant. Maximum projections of z-stacks were generated using FIJI image analysis software.

### Acute hippocampal slice whole-cell patch-clamp recordings

**Figure 3:**
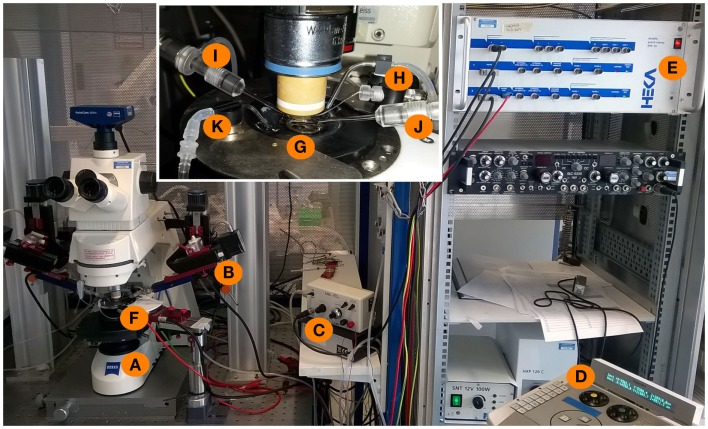
**Experimental setup for whole-cell patch-clamp measurements in acute brain slices**. An upright microscope **(A)** is placed on a *X*–*Y* table and is now freely movable around the bath-chamber **(F)** and the micromanipulators [**(B)**, control unit: **(D)**]. The inset shows a magnification of the bath chamber with the perfusion system **(K)** of the bath and a grid holding the brain slice in the middle of the chamber **(G)**. The recording and stimulation electrodes are marked with **(I,J)**, respectively. The recording electrode is connected to a grounding electrode **(H)** as well as to the amplifier **(E)**. The stimulation electrode is connected to the stimulator **(C)** which is triggered by the amplifier with a 5 mV stimulus command.

**Figure 4:**
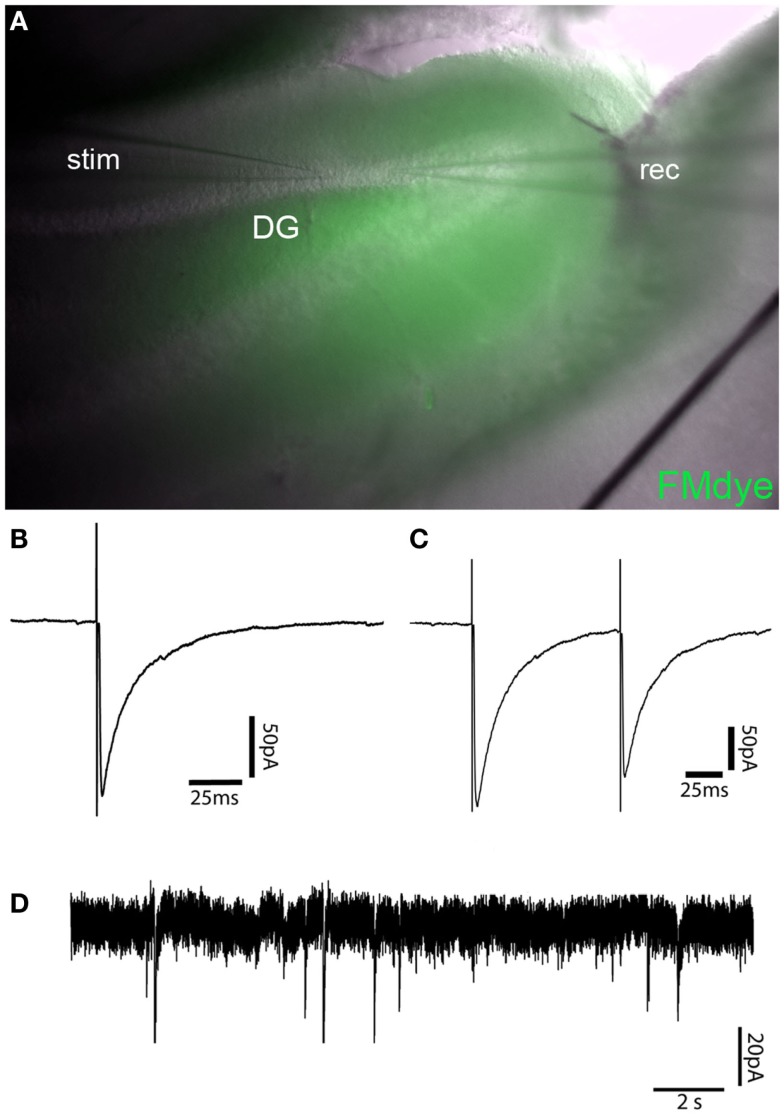
**Recording of GABAergic currents in dentate gyrus granule cell (GC)–basket-cell (BC) synapse**. **(A)** Intravital fluorescent labeling of an IgG injection site in the apex of the dentate gyrus by FM1-43FX coinjection in a 300-μm mouse brain slice 24 h after injection. **(B)** Representative recordings of a single-evoked IPSC from a dentate gyrus GC after electric stimulation of a neighboring BC. **(C)** Paired-pulse depression of IPSCs of the same cell as shown in B with an interpulse interval of 100 ms. **(D)** Trace showing mIPSCs in a dentate gyrus GC after injection of control IgG.

Separate groups of mice were used for *ex vivo* acute brain-slice recording. Twenty-four hours after intrahippocampal IgG injection, mice were deeply anesthetized and decapitated. The brain was removed in ice-cold extracellular artificial CSF (ACSF 1; 40 mM NaCl, 25 mM NaHCO_3_, 10 mM glucose, 150 mM sucrose, 4 mM KCl, 1.25 mM NaH_2_PO_4_, 0.5 mM CaCl_2_, 7 mM MgCl_2_; purged with 95% CO_2_/5% O_2_) and cut in two halves. The bulbus olfactorius was cut in a coronar manner and the brain was glued with the cut face onto the probe-holder with superglue (UHU, Bühl, Germany). Three hundred micrometer thick coronal slices were made with a vibratome (Leica, Wetzlar, Germany; Leica VT1200 S) with an amplitude of 1 mm and a velocity of 0.4 mm/s. Slices were transferred into an incubation beaker with extracellular ACSF suited for patch-clamp recording (ACSF 2; 125 mM NaCl, 25 mM NaHCO_3_, 25 mM glucose, 2.5 mM KCl, 1.25 mM NaH_2_PO_4_, 2 mM MgCl_2_; purged with 95% CO_2_/5% O_2_) and held at 34°C for at least 30 min. For recordings, slices were then transferred into a measurement chamber (Figure 3) superfused with extracellular ACSF 2 with 1.3–1.8 ml/min at room temperature. Recording electrodes pulled from thick-walled borosilicate glass (2.0 mm diameter; Science Products, Hofheim, Germany) were filled with intracellular recording solution containing 140 mM KCl, 10 mM HEPES, 10 mM EGTA, 2 mM Na_2_ATP, 2 mM MgCl_2_, pH 7.2, and an osmolarity of 300–330 mOsmol. Recording electrodes had a final resistance of 3–5 MΩ. Inhibitory postsynaptic currents (IPSCs) were recorded with an EPC10 patch clamp amplifier and Patch-Master Software (HEKA, Lambrecht, Germany). Whole-cell patches were performed of granule cells (GC) held at −70 mV. Recordings were rejected if the resting potential was more positive than −50 mV or changed during the experiments and if series resistance was higher than 20 MΩ. Acceptable membrane capacitance range is from 10 to 30 pF. Evoked IPSCs were isolated by blocking glutamatergic transmission using 10 μM Cyano-Nitroquinoxaline-Dione (CNQX) and 50 μM 2-amino-phosphonovaleric acid (AP-5; Tocris Bioscience, Ellisville, MO, USA). Application of 1 μM tetrodotoxin (TTX; Sigma Aldrich, St. Louis, MO, USA) was used for recordings of miniature IPSCs (mIPSCs). Monosynaptic IPSCs were evoked as described previously (14, 20). Briefly, GABAergic synaptic inputs in whole-cell clamped GCs were evoked by another glass electrode filled with extracellular ACSF 2 that has been placed nearby a neighboring CG located toward the hilar region using a stimulus isolation unit (Isoflex, A.M.P.I, Jerusalem, Israel) (Figures 3 and 4). In this configuration, GABAergic afferents arising from GABAergic basket cells (BC) located at the hilus border region are stimulated. Here, we applied pulses of 200 μs duration at 0.3 Hz while measuring IPSCs of the postsynaptic clamped GCs. Paired-pulse recordings were obtained by stimulating the neurons with an inter-stimulus interval of 100 ms. Recordings were filtered at 2.9 and 10 kHz using the filters of the amplifier. Traces were analyzed by Igor Pro Software (Wavemetrics, Lake Oswego, OR, USA).

## Results

### Trypan blue injections show labeling of the hippocampal formation after stereotactic injection

For direct *ex vivo* verification of the distribution of injected substances within the mouse brain, we injected trypan blue solution at different stereotactic coordinates (Table 1) into the hippocampal formation of both hemispheres (Figure 1). The distribution of trypan blue is shown in Figure 2A. The trypan blue labeling was apparent in most parts of the hippocampal formation including the dentate gyrus and major parts of the CA-regions. In this example, the injection channel can also be identified. This example also demonstrates that only minor lesions of the cortical and hippocampal tissue result by the injection procedure using thin glass pipettes. Beside a slight diffusion of trypan blue along the lateral ventricle, brain compartments surrounding the hippocampus were not labeled.

### Detection of human IgG deposition after stereotactic intrahippocampal injection

Deposition of human IgG was tested 1–3 days after stereotactic injection by using fluorescence coupled commercial antibodies directed to human IgG. Immunohistology was performed in brain slices after perfusion and fixation. Detection of the 10 μg injected human IgG fractions with high titers of anti-GAD65 aAB resulted in exclusive hippocampal immunoreactivity (Figure 2B). Neuronal dendrites in the injection site showed synaptic staining (Figures 2C,D). Magnification of the dendrites as shown here by high-resolution confocal microscopy demonstrates that detailed morphological analysis and quantifications of dendritic structures including spines and synaptic spots are possible (Figure 2D). This method can also be combined with co-immunostaining of further synaptic markers. In comparison to the trypan blue stainings, the overall diffusion of the injected antibodies seems to be less widespread. This could be due to decreased concentrations of injected aAB in the more distant areas of the injection site, absorption of anti-GAD65 aAB by antigens near the injection site, or due to the higher viscosity of the IgG solution.

### Electrophysiological recordings and gabaergic synaptic transmission in the perforans-path – granule cell synapse is not affected by stereotactic IgG injection

Simultaneous injection of patient IgG fractions together with FM1-43FX-Dye allows precise control of the injection site in vital *ex vivo* acute brain slice preparations. FM1-43FX-Dye is fluorescent in contact with cellular membranes and gets incorporated by activity-dependent synaptic vesicle endocytosis. Therefore, the area of IgG injection can be determined in the vital hippocampal slices by localizing the FM1-43FX fluorescence (Figure 4A). The brain slices in this fluorescence labeled area seemed healthy without signs of swelling or tissue destruction. The neurons inside that region showed a healthy shape.

Electrophysiological measurements of IPSCs in GCs also showed reliable vitality in mice injected with control IgG fractions without specific antineuronal reactivity together with FM1-43FX dye. GC had the expected values of resting potential, input resistance, and membrane capacity. Figures 4B,C show averaged sample traces of evoked GABAergic IPSCs after single and paired-pulse stimulation of the perforant path with a time interval of 100 ms. The GABAergic currents in FM1-43FX-injected brain slices had similar amplitudes of 145 pA (mean of 30 sweeps) and kinetics (rise time = 1.27 ms; decay time = 20.05 ms) as in untreated animals. The paired-pulse experiments revealed a significant depression of IPSCs after the second pulse due to the expected short-term plasticity dependent on incomplete vesicle recycling 100 ms after the first induced IPSC (ratio A2/A1 = 0.84). This is in line with published findings on IPSC depression in normal brain slices after extracellular stimulation or in paired recordings of GCs and BCs (14, 23). Recordings of mIPSCs in the control IgG injected brain slices show mean amplitude of 24 pA with a frequency of 0.41 Hz in a similar magnitude as mIPSCs of untreated animals (Figure 4D).

## Discussion

The *in vivo* stereotactic intrahippocampal injection of small volumes offers several advantages in comparison to previously used passive-transfer methods of human IgG samples. First, this method is target-specific for the hippocampal formation as we could demonstrate by trypan blue staining and immunohistology detecting human IgG depositions. This selective delivery of IgG allows investigation of direct aAB effects on hippocampal function, e.g., learning and memory without interference of aAB-induced pathomechanisms in other regions of the CNS. Second, the surgical procedure is fast, and after completing the injections, animals are not affected by any implanted material as it is the case for chronic application of IgG by intraventricular administration using osmotic pumps (24) or by repetitive application using implanted intrathecal (20, 21) or intraventricular (15) catheters. Third, the required amounts of rare human IgG samples are much lower as compared to the other passive-transfer techniques. Moreover, the tissue destruction due to the injection procedure using glass capillaries with thin taper is clearly less compared to implantation of intracerebral catheters.

The parallel application of FM1-43FX fluorescence dyes makes this procedure exceptional suited for *ex vivo* brain slice recordings. Injection sites can be easily identified using a conventional fluorescence light source on the recording setup. As mentioned, tissue destruction is minimal and extracellular as well as patch-clamp recordings can be performed without limitations in regions that have been visually identified by FMdye staining. However, one has to take into account that there might be a difference in the deposition of IgG and FMdye staining. Comparison of immunohistochemical detection of injected human IgG and fluorescent FMdyes are recommended in pilot experiments to test the co-distribution of FMdye and IgG in the injected brain areas. Example recordings shown here demonstrate that GABAergic pathways, e.g., the monosynaptic transmission from BC located in the hilus projecting to dentate gyrus GC can be investigated without any differences in parameters of evoked IPSC and mIPSC as compared to recordings obtained from sham animals that were not injected (20). Here, we used six to eight-week-old mice for injection and recording. In principle, also mice older than 8 weeks can be used in this procedure. Please note that patch-clamp neurophysiology may be more difficult in older animals due to more rigid connective tissue.

Certainly, there are also limitations of this procedure. Implantation of osmotic pumps or intrathecal catheters allows continuous or easy repetitive application in the subarachnoidic space or into the brain parenchyma. Here, repetitive injections can be performed in principle but animals need to be anesthetized again. There is no need for additional trepanation but the skull has to be exposed and skin has to be opened and closed again which increases risk of infections. Further, optimal concentrations of IgG solutions are not determined and can only be varied within the small injection volumes. Preliminary experiments may be necessary to obtain a dose-response curve. It is possible to inject up to 3 μl of IgG into one hemisphere (for example, three separate holes, 1 μl into each) without relevant tissue destruction. Another possibility is to inject 2 μl of IgG per hemisphere with repeated injections of IgG every day over total 3 days. The IgG concentrations can range from 2 to 10 mg/ml IgG. Even higher concentrations of IgG may be tested until clogging of the pipette tip occurs due to the increased viscosity of the solution.

Immunohistochemistry detecting human IgG may give first information of the magnitude and extent of intraparenchymal IgG deposition. Insufficient volume or less IgG concentration may result in only partial application in small areas in the hippocampus. To ensure adequate concentration and allocation of IgG solutions in most of the hippocampal compartment, at least two injection sites, e.g., in the CA3 region and in the dentate gyrus should be selected. As we tested, IgG deposits can be identified up to 1 week after injection. We did not observe any obvious immune cell infiltration or activation.

Together, we here describe a method of reliable, target-specific IgG application into the hippocampal compartment. This is suited for subsequent electrophysiological and histological analyses as an additional and complementary method of passive-transfer applications in mice. With this procedure, direct aAB-induced alternations of neuronal and synaptic activity can be investigated *in vivo* and in slice preparations. In case of aAB to GAD65, inhibitory transmission can be evaluated by recording the BC–GC synapse. In addition, this procedure can also be applied for investigation of aAB-induced dysfunction of excitatory transmission, e.g., induced by aAB to the NMDA- or AMPA receptor. For testing glutamatergic transmission, stimulation of perforant path fibers and recording in GC or stimulation of Schaffer collaterals and recording from CA1 pyramidal neurons would be suitable. Moreover, field potential measurements for analysis of long term potentiation after IgG injection can be performed to obtain information about plasticity changes induced by aAB.

## Conflict of Interest Statement

The authors declare that the research was conducted in the absence of any commercial or financial relationships that could be construed as a potential conflict of interest.
